# Feasible low bone density condition for assessing bioactivity in *ex-in vivo* and *in vivo* studies

**DOI:** 10.1590/1678-7757-2022-0411

**Published:** 2023-06-27

**Authors:** William Phillip Pereira DA SILVA, Leonardo Alan DELANORA, Barbara Ribeiro RIOS, Stéfany BARBOSA, Maria Eloise de Sá SIMON, Cortino SUKOTJO, Leonardo P FAVERANI

**Affiliations:** 1 Universidade Estadual Paulista Faculdade de Odontologia de Araçatuba Departamento de Diagnóstico e Cirurgia São Paulo Brasil Universidade Estadual Paulista (UNESP), Faculdade de Odontologia de Araçatuba, Departamento de Diagnóstico e Cirurgia, São Paulo, Brasil.; 2 University of Illinois at Chicago College of Dentistry Department of Restorative Dentistry Chicago Illinois United States University of Illinois at Chicago, College of Dentistry, Department of Restorative Dentistry, Chicago, Illinois, United States.

**Keywords:** Osteoporosis, Animal models, Bone remodeling, Aging

## Abstract

**Objective:**

To choose a critical animal model for assessments of bone repair with implant installation by comparing senile rats (SENIL) to young ovariectomized rats (OXV).

**Methodology:**

For the ex-in vivo study, the femurs were precursors for bone marrow mesenchymal stem cells. Cellular responses were performed, including cell viability, gene expression of osteoblastic markers, bone sialoprotein immunolocalization, alkaline phosphatase activity, and mineralized matrix formation. For the in vivo study, the animals received implants in the region of the bilateral tibial metaphysis for histometric, microtomography, reverse torque, and confocal microscopy.

**Results:**

Cell viability showed that the SENIL group had lower growth than OVX. Gene expression showed more critical responses for the SENIL group (p<0.05). The alkaline phosphatase activity obtained a lower expression in the SENIL group, as for the mineralization nodules (p<0.05). The in vivo histological parameters and biomechanical analysis showed lower data for the SENIL group. The confocal microscopy indicated the presence of a fragile bone in the SENIL group. The microtomography was similar between the groups. The histometry of the SENIL group showed the lowest values (p<0.05).

**Conclusion:**

In experimental studies with assessments of bone repair using implant installation, the senile model promotes the most critical bone condition, allowing a better investigation of the properties of biomaterials and topographic changes.

## Introduction

The bone tissue undergoes changes in its microarchitecture life-long, physiologically due to the impact of body weight, being more expressive in women after menopause due to estrogen depletion.^1^ Thus, molecular damage adds up to a drastic drop in tissue-specific progenitor cells, and there is a functional decline in tissue homeostasis, which may lead to osteopenia or osteoporosis,^2^ defined as “a systemic skeletal disease characterized by low bone mass and complaint in the microarchitecture of bone tissue, with consequent increase of the bone fragility and susceptibility to fracture.”^3^

The incidence of osteoporosis has increased exponentially, reaching an average of 150 million people worldwide.^1^ Therefore, it is important to choose an osteopenic animal model to evaluate new treatments, prevention, or intervention for osteopenia. Moreover, it is an FDA requirement that these therapies have their efficacy proven in a small and large animal models with known intracortical bone remodeling.^4^

Today, studies that evaluate the properties, quality, and metabolic responses of the most diverse biomaterials have been using the osteoporotic animal study model due to the possibility of evaluating the adaptation, regeneration, stability, preservation, duration, and bioactivity of the material to be tested in an organism with critical metabolic response.^5,6^ The use of rats, mainly R. Norvegicus, Wistar rats, and Sprague-Dawley rats in these types of studies is consolidated.^7,8^

In the case of *in vivo* experimental models with the properties of critical bone, the animal characterized by advanced age is the pioneer, receiving the name of senile. This animal is classified as being aged 15-18 months.^9^ With aging, in addition to estrogen deficiency, molecular and cellular damage is accumulated and associated with the loss of specific tissue progenitor cells, generating a functional decline in tissue homeostasis, which manifests itself as osteopenia/osteoporosis. Thus, bone physiology of senile rats is close to those found in women aged 48-55 years.^5,7,10^

A well-established model for inducing osteoporosis found in the literature is ovariectomy (OVX model).^7^ The rats are subjected to removal or ligation of the ovaries, and after 30 days, the depletion of estrogen levels is consolidated;^7^ this implies an imbalance of bone turnover, in addition to reducing osteoblastic longevity, resulting in the prevalence of osteoclastic activity and bone resorption, leading to rapid loss of trabecular bone mass and osteopenia.^11,12^

In bioactivity studies related to biomedical implants used to replace lost teeth or fixation of craniofacial or orthopedic fractures, different coatings methods have been shown to promote significant attraction of cells of the osteoblastic lineage and accelerate biological responses of mineralization bone. This is clinically justified in bones of low quality and density, such as conditions of osteoporosis, diabetes, irradiation, and osteonecrosis. Therefore, for these analyses, obtaining a more critical model allows the other biases of local or systemic compensations, that may interfere with the peri-implant bone tissue’s biological response, to be eliminated.

The rat tibia for the bone repair assessment was used due to its resemblance to the microarchitecture of human alveolar bone, as reviewed by Glösel, et al.^13^ (2010). It is even more similar to human alveolar bone than rat jaws in terms of bone density, besides being extensively used in the literature.^14-19^

Thus, this study aimed to investigate the properties of critical bone in the main study models of osteoporotic animals found in the literature, comparing the animal model of senile rats and ovariectomized rats through the physiological responses obtained by cell culture (*ex-in vivo*) and installation of osseointegrated implants (*in vivo*).

## Methodology

The project was approved by the Ethics Committee on the Use of Animals of the Faculty of Dentistry of Araçatuba, from São Paulo State University (FOA/UNESP) under protocol #01040-2016. In this study, 69 Wistar rats (Rattus Novergicus) were used, weighing from 300 to 350 grams. For all surgical procedures, the animals were anesthetized, via intramuscular injection, with ketamine hydrochloride (70mg/Kg, Francotar, Virbac, Brazil) and xylazine hydrochloride (10mg/Kg, Rompum, Bayer, Brazil).

### Experimental groups

The 69 rats were randomly assigned to three experimental groups, which were further subdivided in *ex-in vivo* (n=9) and *in vivo* (n=60). These were the experimental groups:

SENIL Group: Senile rats, 18 months old.

OVX Group: Female rats, six months old, subjected to ovariectomy.

SHAM Group: Rats, six months old, subjected to fictitious surgery.

Each group presented n=3 for the *ex-in vivo* study and n=20 for the *in vivo* study.

**Figure 1:**
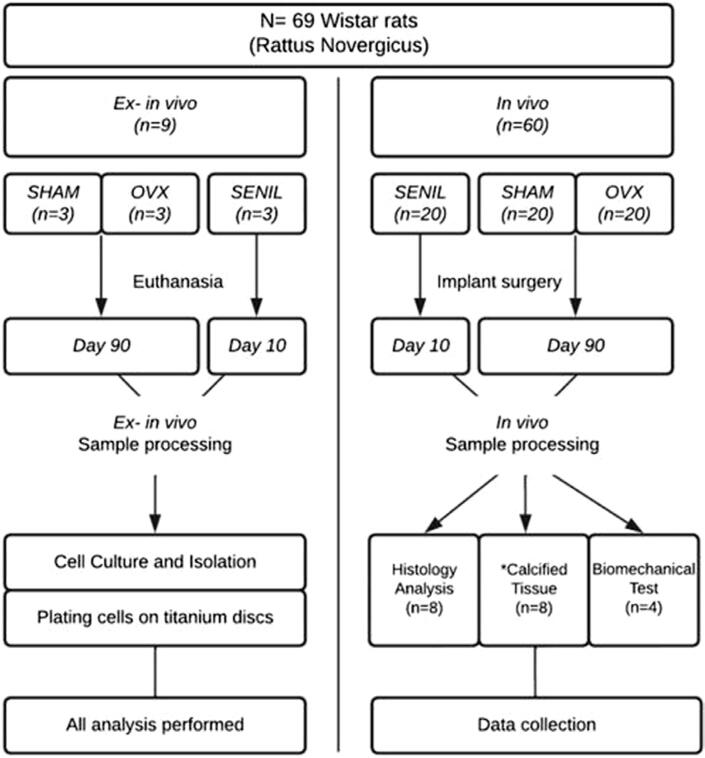
Representation of the entire experimental design, with number of samples and periods. The “*” represents MicroCT analysis, Confocal Laser Microscopy, and Histometry

The experimental design is represented in a flow chart in Figure 1.

### Animals Surgery

A total of 46 young adult rats (*in vivo* n=40 and *ex-in vivo* n=6) were randomly assigned to OVX group (bilateral ovariectomy)^20^ and SHAM group (fictitious surgery). The protocol for the ovariectomy procedure was followed as described by Polo, et al.^14^ (2020). Sodium Dipyrone 500 mg/kg and prophylactic antibiotic therapy with Pentabiotic 0.1 mg/kg of weight were administered in a single intramuscular dose each.

The animals from the three groups were euthanized (corresponding to the animals for the *ex-in vivo* stage) and the other animals were subjected to the implant installation (for the *in vivo* stage). For the serum dosage of estrogen (Estradiol - ng/mL), four animals from each group were sedated and euthanized by decapitation for blood collection, which was processed using the ELISA method.^14^ The measurement kit was the ELISA for rats (Wuhan Fine Biotech CO. Wuhan, China, Cat. No. ER1507). The results of the estrogen measurement showed the following values: OVX Group - average: 8.33±2.18 ng/mL. SHAM Group - average: 58.19±19.00 ng/mL. SENIL Group - average: 22.26±9.31ng/mL. With a statistically significant difference between the SHAM group and the others (p<0.001).

### Ex-*in vivo* study

#### Cell culture and isolation

After 90 days of the fictitious surgery or ovariectomy (SHAM and OVX) and 10 days after the setting of the senile rats in the vivarium (SENIL), the animals were euthanized via anesthetic overdose using sodium thiopental (150 mg/kg; approximately 45-52.5 mg, considering animals weighing from 300-350 mg), by intraperitoneal injection. The femurs were removed and transported in a culture medium containing essential alpha-minimal medium (α-MEM) (Gibco-Life Technologies, USA) with the supplementation of 500 µg/mL of gentamicin and three µg/mL of fungizone.^21,22^ In laminar flow, the femoral bone marrows were extracted by irrigating the growth media (non-differentiating condition) containing α-MEM supplemented with 10% fetal bovine serum (Gibco-Life Technologies), 50 μg/mL gentamicin, and 0.3 μg/mL fungizone.

Bone marrow mesenchymal stem cells (MSC-BM) were grown in a cell culture flask (75 cm^2^) (Corning Incorporated, USA) for seven days, changing the culture medium every 72 hours (37°C), in a humidified atmosphere containing 5% CO_2_ and 95% atmospheric air, changing it three times per week.

#### Plating cells on titanium discs

The cells were trypsinized and plated on sterile Ti-6Al-4V alloy discs that were 10 mm in diameter and 2 mm thickness provided by Emfils Implantes Dentários (Itu, São Paulo, Brazil), with textured surfaces (SLA) according to the parameters commercially available by the company (nitric acids, detergent neutral, 95% alcohol, drying, blasting with aluminum oxide, 99% alcohol, acid nitrate, neutral detergent, distilled water, 95% alcohol, and packaging). The topographic characterization for the SLA implants was assessed by our previous studies,^14,15^ which was the same for this study. The discs were arranged in polystyrene plates with 1.8 mL of modified α-MEM (360 mL α-MEM, 40 mL fetal bovine serum, 2 mL gentamicin, 500 μL fungizone, 4 mL dexamethasone, 4 mL β-glycerophosphate (Sigma-Aldrich), and ascorbic acid (Gibco-Life Technologies), together with the cell concentration according to each analysis to be performed.

#### Cell Growth and Viability

For analysis at three, seven, and 10 days, cells were plated on four discs (2×10^4^ cells per disc). Through the assay of “3(4,5-dimethylthiazol-2-yl)-2,5-diphenyltetrazolium” (MTT, Sigma-Aldrich), after incubation of the cells with 100 μL of MTT (5 mg/mL of PBS) at 37°C for 4h, 1 mL of acid isopropanol (0.04 N HCl in isopropanol) added to each well, stirring was carried out for five minutes. Using a 96-well plate with a transparent bottom with opaque walls (Thermo Fischer Scientific), 150 μL of the solution described above was analyzed using an optical density and read at 570 nm in the lQuant plate reader (Biotek, Winooski, VT, USA). The data were expressed as absorbance (n=4).

#### Real-time PCR analysis

On the seventh day, gene expression analysis of bone markers was performed: runt-related transcription factor 2 (RUNX2), Osterix (OSX), Alkaline Phosphatase (ALP), Bone Sialoprotein (BSP), Osteocalcin (OC), and Osteopontin (OPN). Plating was performed on 12 Ti discs (2×10^4^ cells per disc).^23,24^

RNA extraction was performed using the SV Total RNA Isolation System kit (Promega, USA), according to the manufacturer’s specifications for each bone marker. The RNA was subsequently quantified from 1 µL of the sample in the NanoVue device (GE Healthcare, USA), and its integrity was assessed by electrophoresis on a 1.5% agarose gel. The cDNA strand was made from 1 µg of total RNA. This procedure was performed in a Mastercycle Gradient thermocycler (Eppendorf, Germany) by reaction with the reverse enzyme transcriptase using the SuperScript™ III First-Strand Synthesis Systems kit (Invitrogen-Life Technologies). The TaqMan probe system (Invitrogen-Life Technologies) was used in the 7500 Fast Real-Time PCR System (Applied Biosystems, USA) for the PCR reactions that were done in duplicate. The final volume was 10 µL containing 5 µL of TaqMan Universal PCR Master Mix-No AmpErase UNG (2X), 0.5 µL of the TaqMan probes for the genes of interest (20X TaqMan Gene Expression Assay Mix), and 1,125 ng/µL of cDNA. The data were normalized by the constitutive gene β-Actin and calibrated by the Control group (n=3).

#### Alkaline Phosphatase Activity

On the 10th day, plating was performed on four Ti discs (with 1.5×10^4^ cells per disc) to measure the total protein and alkaline phosphatase activity. The release of thymolphthalein was used from the hydrolysis of thymolphthalein monophosphate substrate to indirectly detect ALP activity in cultured cells using a commercial kit (Labtest Diagnóstica, Brazil). A total of 50 µL of thymolphthalein monophosphate was mixed with 0.5 mL of 0.3 M of diethanolamine buffer, at a pH 10.1 for two minutes at 37°C. The solution obtained was added to 50 µL of the fragments obtained from each well for 10 minutes at 37°C. For color development, 2 mL of 0.09 M Na2CO3 and 0.25 M NaOH were added. After 30 minutes, absorbance was measured at 590 nm, and ALP activity was calculated based on a standard curve using thymolphthalein to give a range of 0.012-0.4 mmol thymolphthalein in h/mL. The data were expressed as ALP activity and normalized for the total protein content.^25^

#### Analysis of the formation of mineralized bone similar to nodules

At 21 days after plating the cells on five Ti disks (1.5×10^4^ cells per disc), fixation was performed with 10% buffered formalin for 24 hours at 4°C. After the period, formalin was removed and dehydrated through a graduated series of alcohols. The discs were stained with 2% red alizarin (Sigma) (pH 4.2) for 10 minutes at room temperature. For the measurement of calcium content, 150 µL of 10% acetic acid was placed in each well and gently stirred for 30 minutes. The cell layer was scraped, and the solution obtained was transferred to a 1.5 mL Eppendorf tube. The solution was vortexed for 30 seconds, heated to 85°C for 10 minutes, and cooled on ice for five minutes. For 15 minutes, the solution was centrifuged (20,000 g) and the supernatant was transferred, adding 40 µL of 10% ammonium hydroxide. The plate was read using the μQuant device (Bio-Tek Instruments.) and the data were expressed as absorbance (405 nm).

## In *vivo* study

### Implant placement

After 10 days of acclimatization of the SENIL rats and 90 days after ovariectomy and sham surgery in the OVX and SHAM groups, respectively, the rats were anesthetized and subjected to bilateral tibial implant placement. Ti-6Al-4V alloy implants with 4 mm diameter and 2 mm thickness were supplied by Emfils Dental Implants (São Paulo - SP, Brazil) with surface texturing (sandblasting and acid etching-SLA) according to the company’s standards. Each animal received an implant in each tibial metaphysis.^14,16,17^

For the placement of the implants, both tibial areas were incised until the periosteum exposed the metaphysis region. The cavitation was prepared using a 1.4-mm diameter drill in a handpiece operating at a speed of 800 rpm under abundant saline solution irrigation. The implants were installed using a manual key with 1.2 mm diameter.

### Histological analysis

For the preparation of the slides, the rats were euthanized using an anesthetic overdose of sodium thiopental (150 mg/kg; approximately 45-52.5 mg, considering animals weighing from 300-350 mg) by intraperitoneal injection at 42 days after the installation of the implants. The tibiae and implants were fixed in formaldehyde and decalcified in EDTA solution (10%) for eight weeks. After that, the implants were removed with a hexagonal digital key of 1.2 mm with counterclockwise movement. The samples were included in paraffin to be cut in a microtome, obtaining histological blades with 5 μm of thickness. These histological slides were photomicrographed at 12× and 40× objectives and used to assess parameters of quality and maturation of newly formed bone tissue.

### Computed Microtomography (MicroCT)

The animals used to analyze calcified tissues were euthanized at 60 days after implant placement. The tibia was removed, subsequently reduced, and stored in 70% alcohol. It underwent analysis by X-ray beam scanning in SkyScan microtomography (SkyScan 1176 Bruker MicroCT, Aatselar, Belgium, 2003) with a Copper and Aluminum filter and a rotation step of 0.05 mm, in 8 μm thick sections (90Kv and 111μA). The images were reconstructed using the NRecon program (SkyScan, 2011; Version 1.6.6.0), and the area of interest was determined.

A circular area was defined around the entire implant (ROI), delimited by 0.25 mm beyond each implant’s surface, using the CTAnalyser-CTAn program (2003-11SkyScan, 2012 Bruker MicroCT Version 1.12.4.0). This area is defined as Total Area (0.25 mm 50 margins around the implants - ROI 2.38 mm × 2.38 mm). Thus, parameters were obtained regarding the amount (BV.TV = percentage of bone volume) and quality of bone tissue (Tb.Th=bone trabecular thickness; Tb.N=number of trabeculae; Tb.SP=separation of bone trabeculae; and To (tot) = percentage of total porosity).

### Biomechanical test of implants

For the biomechanical analysis after fixing the tibia, a 1.2 mm hexagonal digital key was adapted to the implant hexagon and later coupled to the digital torque wrench. An anti-clockwise movement was applied, increasing the reverse torque until the rotation of the implant inside the bone tissue completely separates the bone/implant interface, at which point the torque wrench registered the maximum torque peak in Newton per centimeter (N.cm.).

### Confocal laser microscopy (peri-implant bone dynamics)

On days 14 and 42 after implant placement, the animals received the administration of calcein and alizarin intramuscularly, respectively, at a dosage of 20 mg/kg of the animal, to carry out the analysis in laser confocal microscopy, investigating the dynamics of the bone.

After microtomography, the samples were subjected to Technovit resin embedding by the Exakt processing protocol (Cutting System, Apparatebau, Gmbh, Germany). Laminae with approximately 80 μm thickness were made by the cutting system and in an automatic polishing machine, as described by Momesso, et al.^15^ (2020).

The analysis was performed in a Leica CTR 4000 CS SPE laser confocal microscope (Leica Microsystems, Germany), obtaining images with 1×1 mm^2^, corresponding to optical sections of 512×512 pixels. A total of 30 slices were made, obtaining a 56-µm scan.

The images were analyzed using ImageJ program (Processing Software and Image Analysis, Canada). A threshold tool was used to measure the fluorochromes area standardizing in hue, saturation, and brightness, highlighting the green fluorescence (calcein precipitation), followed by red fluorescence (alizarin red precipitation).

### Histometry

After using the confocal microscopy assessment, the blades were washed in deionized water and stained with alizarin red and Stevenel blue. The histological slides were collected, and the files were saved as TIFF files and transferred to the Image J program (Processing Software and Image Analysis, Ontario, Canada), to evaluate the bone/implant contact area (BIC) and bone neoformation area (BNF).

Using the “free hands” tool, it was possible to define and measure the BIC between the newly formed bone tissue and the implant surface. The BNF was evaluated in the most central implant thread of each implant.

## Statistical analysis

For the real-time PCR data, the Kruskal-Wallis test was applied. The one-way ANOVA test was performed in the data obtained through the Alkaline Phosphatase Activity and Mineralization Nodes. In the analysis of Cell Viability the Two-way ANOVA test was used. For the data by microtomography, the T-test was performed. For the torque-reversal analysis, the Mann-Whitney test was used. In the analysis of peri-implant bone dynamism by Calcein and Alizarin, the Kurskal-Wallis test was applied. The results obtained by histometry (BIC and BNF) were analyzed using a One-way ANOVA test. All results were subjected first to the normality test (Shapiro-Wilk), with a significance value of p<0.05, using SigmaPlot 12.0 program (Exakt Graphs and Data Analysis, San Jose, California, USA).

## Results

### *Ex-in* vivo

#### Cell growth and viability

**Figure 2:**
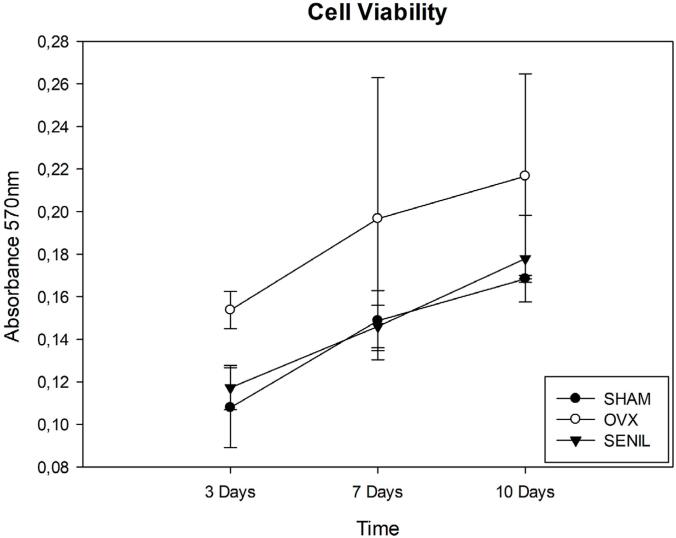
Graphic image of cell growth and viability for SHAM, OVX, and SENIL groups, respectively at 3, 7, and 10 days: SHAM (0.1078±0.0187), (0.148±0.014), (0.168±0.001); OVX (0.153±0.008), (0.196±0.066), (0.216±0.048); and SENIL (0.117±0.010), (0.146±0.010), (0.177±0.020)

The representative values attributed in the analysis of cell growth and viability were separated by groups of animals according to the proposed times, and all groups obtained an increase in growth and gradual cell viability from three to seven days (p>0.05) and from seven to 10 days (p>0.05). On the third day, the SHAM group’s culture showed more critical proliferation compared to the others, among which there was no statistically significant difference. On the seventh day of culture, the SENIL group obtained a lower result, followed by OVX, but there was no statistical significance between them. On the 10th day, cell proliferation remained lower in the SENIL group (p>0.05) (Figure 2).

#### Real-time PCR analysis

**Figure 3:**
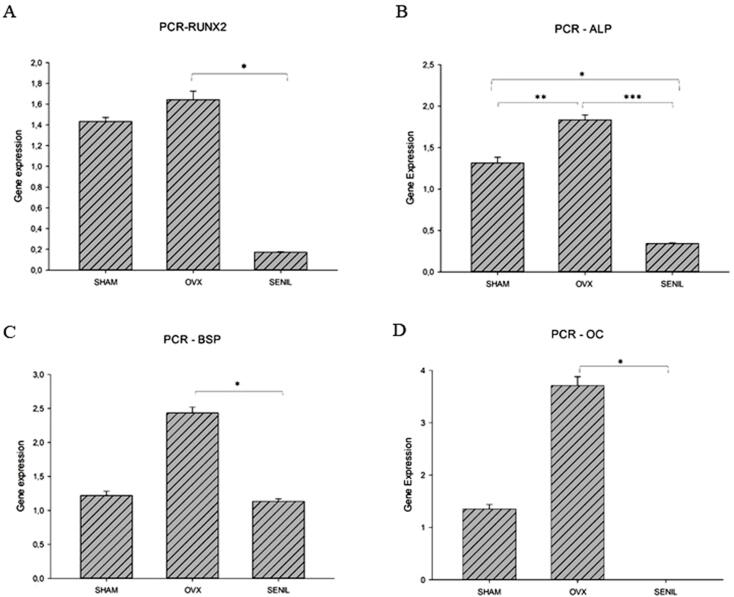
(A) Graphic image of gene expression by real-time PCR of RUNX2 – SHAM (1.432±0.038), OVX (1.642±0.084), and SENIL (0.171±0.004). *Showing statistical difference between OVX and SENIL (p<0.001). (B) Graphic image of gene expression by real-time PCR of the expression of ALP – SHAM (1.312±0.071), OVX (1.834±0.059), and SENIL (0.341±0.008). * Showing statistical difference between SHAM and SENIL (p<0.001); ** statistical difference between SHAM and OVX (p<0.001); and *** statistical difference between OVX and SENIL (p<0.001). (C) Graphic image of gene expression by real-time PCR of the expression of BSP – SHAM (1.219±0.064), OVX (2.434±0.085), and SENIL (1.132±0.038). * Showing statistically relevant results between OVX and SENIL (p<0.001). (D) Graphic image of gene expression by real-time PCR of the expression of OC – SHAM (1.351±0.086), OVX (3.707±0.170), and SENIL (0.002±0.0008). * Showing statistical difference between OVX and SENIL (p<0.001)

The real-time PCR analysis showed that gene expression was lowest in all analyzed proteins for the SENIL group when compared with the OVX group (p<0.05). When comparing the SENIL group with the SHAM group, there was a statistical difference only in the ALP protein (p>0.05); the other proteins showed similar results (Figure 3).

#### Alkaline phosphatase activity

**Figure 4:**
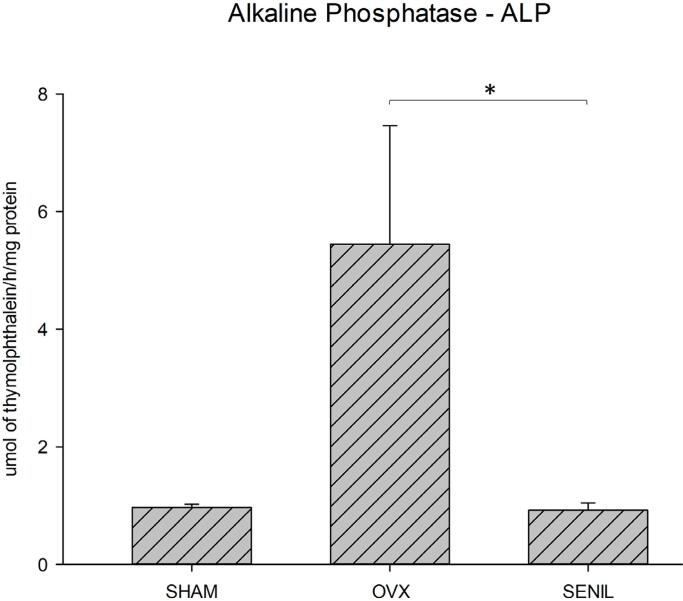
Graphic image of the activity of Alkaline Phosphatase (ALP) in 10 days for the SHAM (0.9677±0.0566), OVX (5.4458±3.8278), and SENIL (0.9208±2) groups, 6525). *Showing statistical difference between OVX and SENIL (p<0.001)

In the analysis of alkaline phosphatase, the OVX group showed higher results than the SENIL group, with a statistical difference (p=0.001), and the SHAM group, with no significant difference (Figure 4).

#### Analysis of the formation of mineralized bone similar to nodules

**Figure 5:**
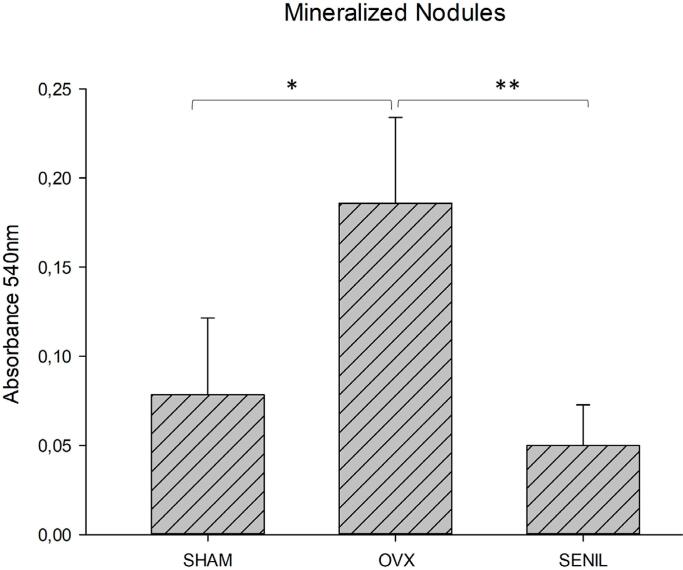
Graphic image of the formation of mineralized bone similar to nodules stained with Alizarin Red from SHAM (0.078±0.039), OVX (0.185±0.043), and SENIL (0.05±0.020) groups. *Showing statistical difference between SHAM and OVX (p<0.001) and ** between OVX and SENIL (p<0.001)

The evaluation of the mineralized nodules confirmed that the SENIL group obtained less mineralization than the others. The OVX group also stood out in this analysis, obtaining a statistical difference when comparing the SENIL group with the OVX and OVX compared to the SHAM (p<0.001) *(*Figure 5).

## In *vivo*

### Histological parameters and biomechanical analysis (reverse torque)

**Figure 6:**
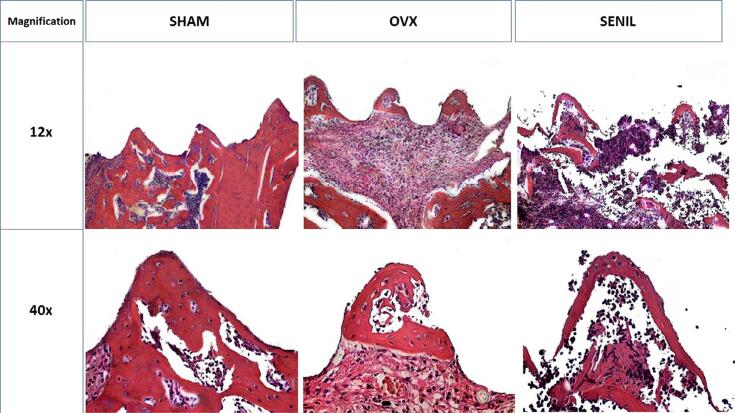
Graph representing the reverse torque parameter of the experimental groups. Photomicrograph of SHAM (4±0.816), OVX (2±1.414), and SENIL (4.3±2.516) in Hematoxylin and Eosin staining in 12× and 40× magnifications for histological analysis

In the qualitative histological analysis, both OVX and SHAM groups presented neoformation of bone tissue in the higher portions (peaks) of the bone/implant interface, while in the lower regions (valleys), they presented a more significant amount of organized connective tissue. In the SENIL group, bone tissue neoformation occurred in the highest portion of the region corresponding to the implant thread, with a high amount of organized connective tissue. (Figure 6).

### Biomechanical Analysis

In the biomechanical analysis between the groups, it was possible to verify more consistent data in the SHAM group (4±0.81 N.cm), with a similarity between them. In contrast, the OVX group (2±1.4 N.cm) showed the lowest values and SENIL (4.33± 2.51 N.cm) the most dispersed values. No group showed a statistical difference between them.

### Confocal laser microscopy (peri-implant bone dynamics)

**Figure 7:**
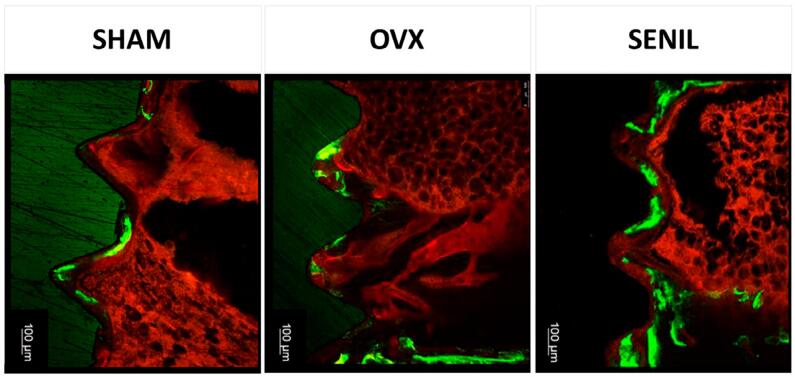
Photomicrograph representing the fluorochromes labeling Calcein and Alizarin in SHAM calcein (0.891±0.573); SHAM alizarin (0.169±0.159); OVX calcein (0.444±0.478); OVX alizarin (0.259±0.326); SENIL calcein (1.973±0.856); and SENIL alizarin (0.617±0.496), followed by the graph corresponding to the same parameter

In the analysis of fluorochromes by confocal microscopy, the SENIL group showed much higher values regarding the old bone (calcein/green) compared to the other experimental groups, presenting statistical differences from the SENIL group to the other groups. In the analysis of alizarin, the groups showed no statistical difference (Figure 7).

### Microtomographic parameters (MicroCT)

**Figure 8:**
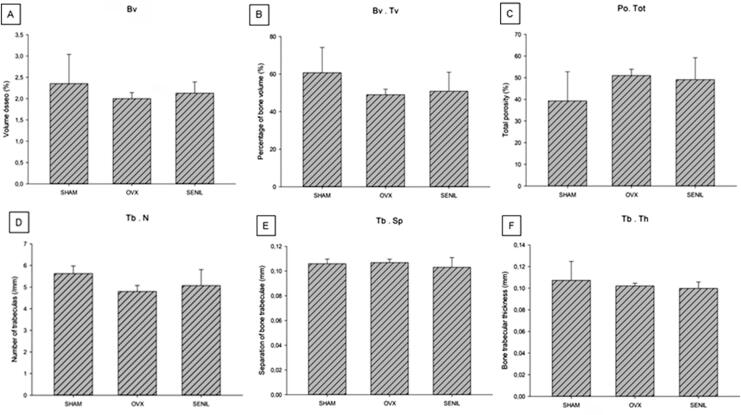
Graphical results of the microtomographic parameters: A) Bv: SHAM (2.352±0.686), OVX (1.998±0.142), and SENIL (2.128±0.263). B) Bv.Tv: SHAM (60.718±13.458), OVX (49.04±2.950), and SENIL (50.85±10.125). C) Po (tot): SHAM (39.281±13,458), OVX (50.958±2.950), and SENIL (49.127±10.087). D) Tb.N: SHAM (5.627±0.353), OVX (4.801±0.276), and SENIL (5.069±0.741). E) Tb.Sp SHAM (0.105±0.003), OVX (0.106±0.002), and SENIL (0.103±0.007). F) Tb.Th SHAM (0.107±0.017), OVX (0.102±0.002), and SENIL (0.099±0.005). No group showed a statistically significant difference (p>0.05)

The microtomographic parameters were similar for all groups (p>0.05) (Figure 8).

### Histometry

**Figure 9:**
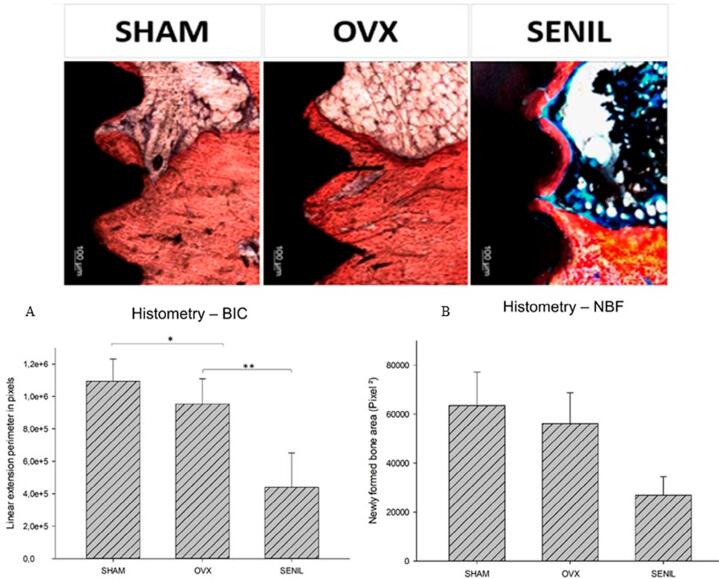
Representative images of the calcified tissue of the experimental groups used for histometric analysis. (A) Graph representing the results of BIC: SHAM (1,093,331.0±137355.1), OVX (953528.2±156013,7), and SENIL (440.199.0±211636), with a statistically significant difference between SHAM-SENIL and OVX-SENIL groups (p<0.05). (B) Graph representing the results of BNF: SHAM (63543.2±13655.67), OVX (56191.22±12626.91), and SENIL (26852.25±7567.436)

As for the histometric analysis, the values assigned to BNF were higher for SHAM and OVX, with a statistically significant difference (p<0.05) compared to SENIL, which had the smallest area of BNF. In the analysis of the BIC, the values followed the same as the BNF, in which the SENIL group had the lowest values, with a statistically significant difference (p<0.05). The SHAM and OVX groups’ values showed no statistical difference (p>0.05) (Figure 9).

## Discussion

The rat is the most frequently used animal in experimental studies as it is easy to manipulate, cheap, and its biological response share a certain similarity with humans.^26^ Therefore, the search for a critical bone model associates the facilitations that the experimental model offers, in addition to the challenging bone characteristics resulting from senility or ovariectomy.^5,6,8^

The use of long bones to evaluate bone properties and osseointegration is widely used in the literature, and bones such as tibia have a microarchitecture and density closer to human maxillary bones than rodents’ own maxillary bones.^14,15,20^ Regarding cell culture for analysis of cell growth and viability, all groups showed cell growth on the tested surface, which shows that the titanium surface was viable for other analyses.^21,22^

In gene expression analysis (PCR) evaluating the chronology of bone repair, all proteins and enzymes evaluated (RUNX 2; Alkaline phosphatase (ALP); Bone sialoprotein (BSP); and Osteocalcin (OC)), referring to the period osteoblastogenesis and mineral deposition, OVX rats stood out when compared with the others (Sham and SENIL groups), and SENIL was more critical for bone repair, representing that rats that had induced osteoporosis (OVX) were more easily differentiated in cells of osteoblastic lineage. Although this experimental model has been induced to osteoporosis, its organism presents effective homeostatic dynamics arising from age and may find a greater facility for physiological compensations. On the other hand, senile animals, in addition to natural osteoporotic condition, present metabolic deficiencies, facing a more significant biological effort.

For alkaline phosphatase activity, this study obtained results similar to those previously mentioned, reaffirming the expression of this enzyme in the initial phase of mineralization. In this analysis, the SENIL group faced greater bone neoformation difficulties. Finally, the formation of mineralized nodules represents numerically and visually the final balance of bone formation. The data obtained with this analysis also corroborates the hypothesis mentioned above, since OVX animals obtained a greater number of mineralized nodules, followed by SHAM and finally by SENIL groups.^9,21,22^

In the analyses related to reverse torque, histology, and confocal microscopy, the SHAM group showed a more significant amount of neoformed bone; as expected, the values for reverse torque were similar to that of the SENIL group, as well as in the analysis of confocal microscopy, in which the two groups showed the closest values compared with the three experimental groups. This suggests that the amount of old bone, marked by the green color in confocal microscopy, may have resulted in the maturation of this bone in contact with the implant, generating greater resistance in the removal of this implant. This disagrees with the OVX group, which had the lowest values of old and new bone, which can be translated as a more immature bone and therefore presented the lowest values in the biomechanical test. Chen, et al.^2^ (2018) also compared these three experimental groups, with different nominations, and the results showed that OVX and SENIL groups presented similar values in many of the tests performed. A particularity is that the age considered senile was 12 months.^7,9,11^

The histometric analysis is worth more attention since it presented lower and statistically different values for the SENIL group. It showed lower values for BNF and BIC in comparison with both OVX and SHAM groups, which means little production of new bone and presence of old bone not absorbed/remodeled. This shows the low quality or deficiency in the critical bone turnover of this group, which is unable to have efficient replacement of old bone and deposition of new bone. From a physiological point of view, this would lead to a more fragile, less resilient bone that is therefore more susceptible to fractures.^27^

The performance of the OVX group similar or even better than the control group has already been presented in other works such as Kim, et al.^28^ (2018). After specific treatment, the bone starts to behave in a similar or even more efficient way in terms of repair. We believe that such behavior is due to the mechanisms of physiological compensation of ovariectomized animals. Additionally, we observed a critical scenario when evaluating the behavior of bone metabolism, both *in vitro* and *in vivo*, of senile animals. This fact is corroborated by other studies such as Chen, et al.^2^ (2018), Toro, et al.^10^ (2019), Thompson, et al.^20^ (1995) and many others, that age is closely associated with the decline in tissue repair.

According to Mosekilde^4^(1995), young organisms have robust bone trabeculae and a good relationship with the medullary portion, while senile animals present remodeling with a slightly negative balance modulated by the action of osteoclasts and resorption of the cortical bone due to cortico-endosteal and intracortical (Haversian) bone remodeling, making this structure even more fragile. Regarding menopause and estrogen depletion associated with decreased bone density, Kimmel^9^ (2001) indicates a relationship between natural menopause and its intermittence gradually increasing estrogen spikes with induced menopause and its hormonal changes. This indicates that the organism of early induced menopause, by removal of the ovaries, may present an adaptive bone response to this condition, different from senile animals, making it the most critical animal model for assessing bone repair.

## Conclusion

The comparison of the experimental groups OVX and SENIL shows that for the evaluation of the tested biomaterial, osseointegrated implants, the osteoporotic model promoted by the SENIL group was the most challenging experimental model. This was characterized by a more significant loss of bone mass and less new bone formation, justified by greater osteoclastogenic activity. This translates to an old bone with deficient turnover, characterizing fragile bone and more critical repair results, which in experimental studies allow better assessment of biomaterials properties and topographic changes in deteriorated bone physiology.
